# Design of novel Surfactant Modified Carbon Nanotube Paste Electrochemical Sensor for the Sensitive Investigation of Tyrosine as a Pharmaceutical Drug

**DOI:** 10.15171/apb.2019.016

**Published:** 2019-02-21

**Authors:** Nagarajappa Hareesha, Jamballi Gangadharappa Gowda Manjunatha, Chenthattil Raril, Girish Tigari

**Affiliations:** Department of Chemistry, FMKMC College, Constituent College of Mangalore University, Madikeri, Karnataka, India.

**Keywords:** Carbon nanotube, Sodium dodecyl sulfate, Tyrosine, Voltammetry

## Abstract

***Purpose:*** The novel sodium dodecyl sulfate modified carbon nanotube paste electrode (SDS/ CNTPE) was used as a sensitive sensor for the electrochemical investigation of L-tyrosine (TY).

***Methods:*** The electrochemical analysis of TY was displayed through cyclic voltammetry (CV) and differential pulse voltammetry (DPV). The surface morphology of SDS/CNTPE and bare carbon nanotube past electrode (BCNTPE) was reviewed trough field emission scanning electron microscopy (FESEM).

***Results:*** The functioning SDS/CNTPE shows a voltammetric response with superior sensitivity towards TY. This study was conducted using a phosphate buffer solution having neutral pH (pH=7.0). The correlation between the oxidation peak current of TY and concentration of TY was achieved linearly in CV method, in the range 2.0×10^-6^ to 5 ×10^-5^ M with the detection limit 729 nM and limit of quantification 2.43 μM. The investigated voltammetric study at SDS/CNTPE was also adopted in the examination of TY concentration in a pharmaceutical medicine as a real sample with the recovery of 97% to 98% .

***Conclusion:*** The modified electrode demonstrates optimum sensitivity, constancy, reproducibility, and repeatability during the electrocatalytic activity of TY.

## Introduction

L-tyrosine (TY) is one of the standard aromatic amino acid found in some foodstuffs and living organisms; it performs as a building block of the proteins.^1-3^ TY is acting as an originator of neurotransmitters, pigments, and hormones such as dopamine, thyroid and melanin.^4-7^ TY facilitates to improve mental performances and attentiveness or remembrance; also TY is use to avoid depression or attention deficit disorder, high blood pressure, Parkinson’s disease, alcoholism, and cocaine addiction and too helpful for photosynthesis. The abnormality of TY could cause albinism, alkaptonuria, low blood pressure, low body temperature and an underactive thyroid.^8^ Some people have the low intensity of TY in their bodies because of the hereditary condition called phenylketonuria due to this body do not develops amino acid called phenylalanine.^9,10^

**Scheme 1:**
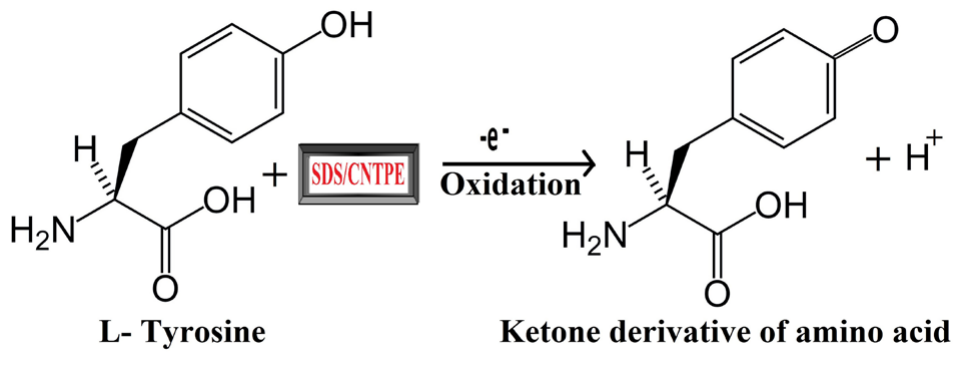
Structure and oxidative reaction mechanism of TY.

In the previous works numerous methods were evidenced, such as chromatographic studies, mass spectroscopic studies,^11-14^ spectrophotometric studies^15,16^ and electrochemical studies for the determination of biologically active scaffolds, in these studies, electrochemical studies^17-22^ such as cyclic voltammetry (CV) and differential pulse voltammetry (DPV) shows excellent sensitivity, and they are trouble-free to handle in the laboratory circumstances, these are used to study the kinetics of a reaction, electron and ion transfer reactions.^23^ Also, these techniques mainly depends on chemically modified electrodes that are working electrodes. Here we use multi-walled carbon nanotube (MWCNT) for the preparation of the working electrode because it shows excellent sensitivity, steadiness, high electrical conductivity and high surface area towards biologically active moieties.^24^ The modification of bare carbon nanotube past electrode (BCNTPE) was done using SDS as an anionic surfactant material; it gives more excellent sensitivity and stability during the adsorption of the analyte^25^ due to the hydrophobic and hydrophilic behavior. Hence the analysis of TY was carried out at sodium dodecyl sulfate modified carbon nanotube paste electrode (SDS/CNTPE). CV and DPV were also used to investigate the reaction mechanism of TY in the pharmaceutical drug as a real sample. The structure and oxidative reaction mechanism of TY at the SDS/CNTPE is shown in Scheme 1.

## Materials and Methods

### Materials

Silicone oil, Sodium hydroxide and SDS were purchased from Nice Chemicals, India. Disodium hydrogen phosphate and Monosodium dihydrogen phosphate were bought from Himedia, India. L-Tyrosine was purchased from Molychem. MWCNT (OD: 30-50 nm and length: 10-30 μm) was bought from Sisco research laboratories Pvt. Ltd. Mumbai.

### Preparation of chemical solutions

The phosphate buffer solution (PBS) was made by adding the suitable amount of 0.2 M monosodium dihydrogen phosphate and 0.2 M disodium hydrogen phosphate. 25×10^-4^ M TY solution was prepared by adding calculated amount of 0.01M NaOH solution. SDS (25×10^-3^ M) solution was made by dissolving an appropriate amount of SDS in distilled water.

### Instrumentation

The CV and DPV methods were performed on a model 201 (EA-201, Chemilink system, Mumbai, India). SDS/CNTPE and BCNTPE were utilized as the working sensors. An aqueous saturated calomel electrode was adopted as a reference electrode and platinum wire as an auxiliary electrode. The computer was used for the data storage and the processing.

### Preparation of sensitive BCNTPE and SDS/CNTPE

The BCNTPE was made by mixing of CNT and silicon oil in the ratio of 60:40 (w/w %) in mortar thoroughly until the homogeneous paste was obtained. The prepared carbon nanotube paste was arranged on the surface of the Teflon tube having a cavity of 3 mm diameter, and the electrical contact was done through copper wire joined to the paste. Similarly, the modified carbon nanotube paste electrode (MCNTPE) was prepared by adding 10 μL SDS solution through immobilization technique.

## Results and Discussion

### SDS concentration variation for the analysis of TY

**Figure 1:**
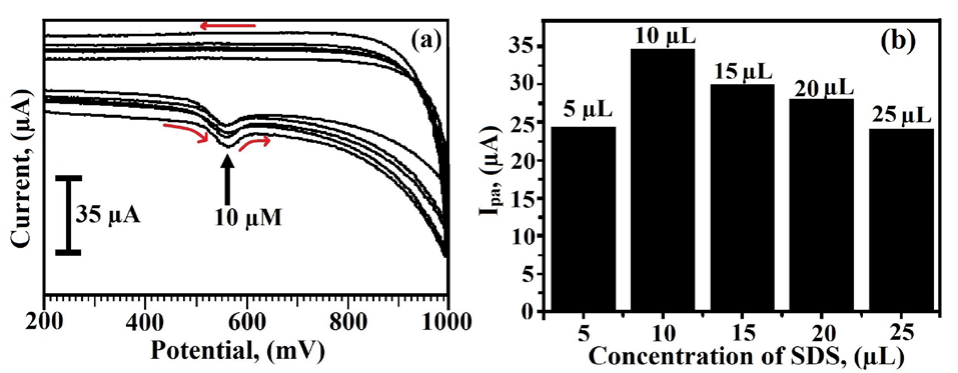
(a) Concentration of SDS variation using 0.2 M PBS of pH 7.0 at the scan rate 100 mV/s. (b) The plot of [SDS] v/s plot of anodic peak current (I_pa_).

The oxidation peak current of 2×10^-4^ M TY was calibrated using CV through the variation of the concentration of SDS at BCNTPE from 5 μL to 25 μL (Figure 1a). The graph was plotted as the concentration of SDS (μL) v/s anodic peak current (I_pa_) (μA) and demonstrates that the anodic peak current value was more negative for 10 μL SDS (Figure 1b), due to the more catalytic action of TY. Hence we utilized 10 μL SDS for the modification of the electrode throughout the experiment.

### Surface morphology of BCNTPE and SDS/CNTPE

**Figure 2:**
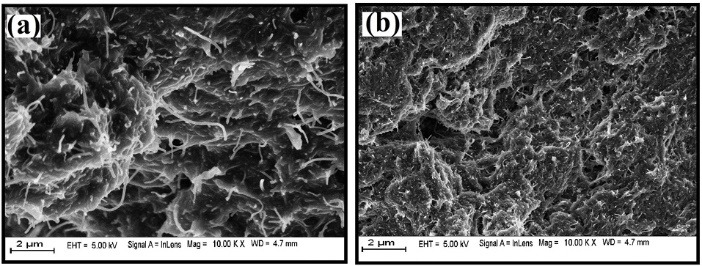
(a) FESEM representation of BCNTPE. (b) FESEM representation of SDS/CNTPE

The surface morphology of BCNTPE and MCNTPE was interpreted through field emission scanning electron microscopy (FESEM). Figure 2a and 2b exhibit the different morphological characters of the BCNTPE and SDS/CNTPE. The FESEM picture accurately shows the tube-like fibers in BCNTPE, whereas in SDS/CNTPE, uniform deposition of SDS surfactant like the spongy arrangement, this confirms that SDS films were modified on the surface of BCNTPE.

### Electrochemical behavior of TY at SDS/CNTPE

**Figure 3:**
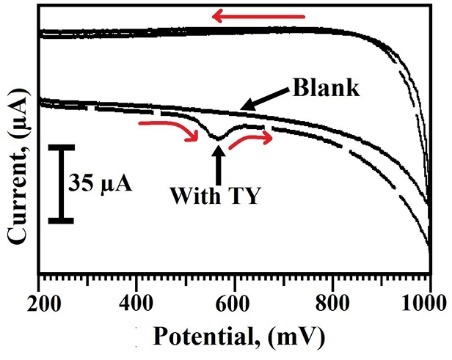
Cyclic voltammograms of SDS/CNTPE with TY (2×10^-4^ M) (dashed line) and without TY (solid line) in 0.2 M PBS of pH 7.0 at the scan rate 100 mV/s.

Figure 3 illustrates the cyclic voltammograms of the electrochemical behavior of TY, it having solid line represents the absence of TY, and dashed line represents the presence of 2×10^-4^ M TY with 0.2 M PBS (pH 7.0) by using CV within the potential scale 200–1000 mV at the sweep rate 100 mV/s at SDS/CNTPE. The solid line shows no peak but the dashed line displays a broader oxidation peak at 569 mV with an oxidation peak current (I_pa_) -34.7 μA, it indicates that the electrochemical development depends on TY only at SDS/CNTPE.

### Electrocatalytic activities of TY at SDS/CNTPE

**Figure 4:**
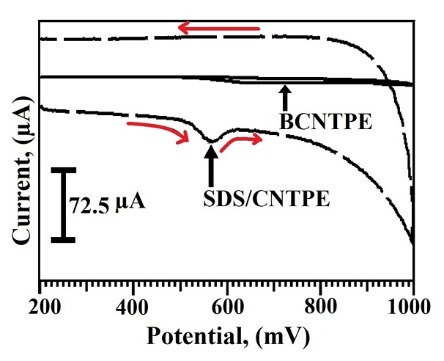
Cyclic voltammograms of 2×10^-4^ M TY in 0.2 M PBS having pH 7.0 at SDS/CNTPE (dashed line) and BCNTPE (solid line) with the scan rate 100 mV/s.

The voltammetric activities of 2×10^-4^M TY in 0.2 M PBS having neutral pH with the scan rate 100 mV/s at BCNTPE and SDS/CNTPE, represented by the dashed line and solid line respectively. The voltammograms (Figure 4) reveals that BCNTPE did not show any electrochemical response compared to the SDS/CNTPE. The oxidation of TY occurs irreversibly at SDS/CNTPE with the higher anodic peak current (I_pa_) of -34.7 μA, and anodic peak potential (E_pa_) of 569 mV, these data of SDS/CNTPE and BCNTPE verifies that the electrocatalytic action of TY was more sensitive at SDS/CNTPE compared to BCNTPE.

### Effect of solution pH on the analysis of TY at SDS/CNTPE

**Figure 5:**
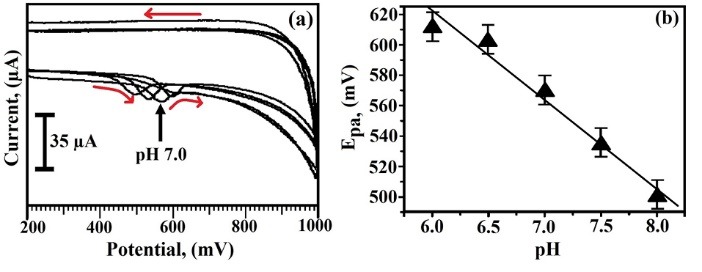
(a) Cyclic voltammograms at SDS/CNTPE in 0.2 M PBS in different pH values from 6.0 to 8.0 containing TY (2×10^-4^ M) with the scan rate 100 mV/s. (b) The plot of the anodic peak potential (Epa) V/s pH at SDS/CNTPE.

The CV technique was used to study the cause of pH in the range of 6.0-8.0 in 0.2 M PBS. Figure 5a shows the voltammetric response of 2×10^-4^ M TY, here the electrode peak potential was driven towards the positive side by increasing the pH and also shows that the pH 7.0 has more current sensitivity with more relevant for electron transfer in TY, hence the pH 7.0 was chosen throughout the experiment. The plot of oxidation peak potential (E_pa_) and the solution pH obtained linearly with linear regression equation E_pa_ (mV) = 969.2–58 pH (R = 0.984) where R is the correlation coefficient (Figure 5b). A slope 58 mV/pH is more close to the theoretical slope value 58.5 mV/pH; this confirms that the proportion of electron and proton concerned in the catalytic reaction was 1:1; hence the oxidation of TY is one electron transfer process.^26^

### Effect of scan rate on the analysis of TY at SDS/CNTPE

**Figure 6:**
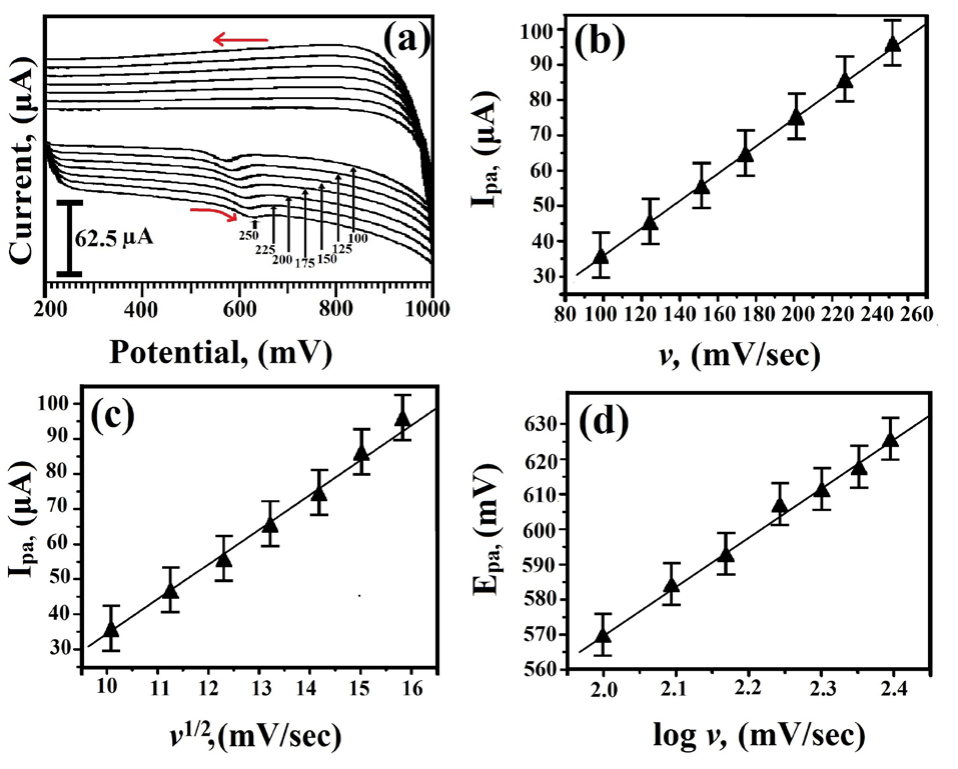
(a) Cyclic voltammograms of TY at SDS/CNTPE in PBS having pH 7.0 at various scan rates from 100 to 225 mV/s. (b) The plot of peak current of TY as function of scan rate. (c) The plot of peak current and the square root of the scan rate. (d) The plot of anodic peak potential v/s log scan rate.

The oxidation peak current of 2×10^-4^ M TY was measured at SDS/CNTPE via CV method with variable scan rates from 100-250 mV/s was shown in Figure 6a and it reveals that as the scan rate (*v*) increases the peak current shifted towards negative range (Considered as positive values). The plot of the anodic peak current (I_pa_) v/s scan rate (*v*) was plotted and had a good linearity (Figure 6b) with a linear regression equation I_pa_ (μA) = 5.83 + 0.402 *v* (mV/s) (R = 0.994). A plot between peak current and the square root of the scan rate in the range of 100 to 250 mV/s was plotted (Figure 6c) and having good linearity between anodic peak current and the square root of scan rate, with a linear regression equation as I_pa_ (μA) = 70.6145 + 10.3009 *v*^1/2^(mV/s) (R = 0.993), indicating that the electron transfer reaction was diffusion-controlled. Also, the plot of E_pa_ and log *v* was plotted (Figure 6d). The plot obtained was having good linearity between anodic peak potential and the log* v*, with a linear regression equation E_pa_ = 70.6145 + 10.3.009 log *v* (R = 0.993), indicating that the electrochemical oxidation reaction continues through the electron transfer mechanism., The irreversible electrode process, E_pa_ and ν were defined on the basis of Laviron’s equation^27^ (1)

(1) $$E_{pa}=E^{0}+\left(\frac{2.303RT}{\alpha nF}\right)\log\left(\frac{RTk^{0}}{\alpha nF}\right)+\left(\frac{2.303RT}{\alpha nF}\right)\log v$$

Where *α* is the charge transfer coefficient, k^0^ is the rate constant of the reaction, *n* is the number of electron transfer, *υ* the scan rate and E_0_ is the standard potential. The slope was equal to 2.303RT/αnF and the value of nα obtained was 0.4279, but for irreversible process, α was assumed as 0.45, hence the oxidation reaction of TY proceeds through one electron (n=1) transferred process.

### Analysis of repeatability, reproducibility, and stability

The CV technique was utilized for the investigation of repeatability and reproducibility of TY at SDS/CNTPE in 0.2 M PBS with pH 7.0 at the scan rate 100 mV/s. SDS/CNTPE shows a good repeatability for 5 successive cycles (2×10^-4^ M TY solution changes for every cycle but SDS/CNTPE kept constant) gives a standard deviation (RSD) of 1.1%. Also, it shows a good reproducibility with constant 2×10^-4^M TY solution for 5 successive cycles (SDS/CNTPE changes for every cycle) gives an RSD of 1.265%. The stability was studied by 40 consecutive cycles and calculated by using percentage degradation formula^28^(2):

(2) $$\text{Percentage degradation}=\frac{I_{pn}}{I_{p1}}\times 100$$

Where I_pn_ is the peak current at the last cycle and I_p1_ is the peak current at the first cycle, here 99% of the initial current signal was regained even after 40 cycles; this data shows that SDS/CNTPE is having excellent stability and sensitivity towards TY solution.

### Effect of concentration of TY

**Figure 7:**
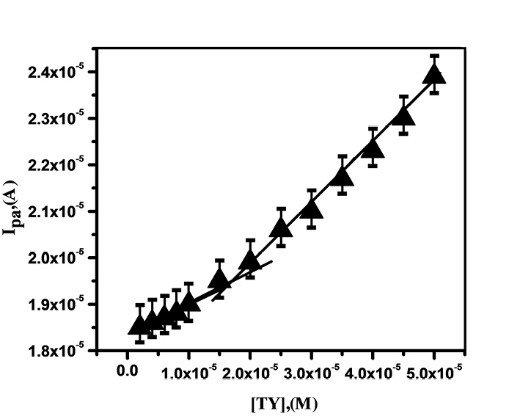
Calibration plot for the determination of TY using 0.2 M PBS of pH 7.0 at the scan rate 100 mV/s at SDS/CNTPE.

Figure 7 was plotted as the concentration of the TY v/s oxidation peak current (I_pa_), provides two linear relationships, in that we considered 2.0×10^-6^ to 5 ×10^-5^ M with a linear regression equation, I_pa_ (A) =1.712 × 10^-5^ + 0.13 C (M) (R=0.9996). The limit of detection (LOD) and limit of quantification (LOQ) for TY were calculated by using the following formulas, LOD=3 S/M and LOQ=10 S/M (where S is the standard deviation of blank and M is the slope of the calibration plot), obtained values of LOD and LOQ were 729 nM and 2.43 µM respectively. The obtained detection limit was compared with some related works were reported in Table 1^5,10,29-32^ and confirms that SDS/CNTPE has nearly comparable sensitivity and nearer limit of detection.

**Table 1:** Comparison of different detection limit values with the detection limit value of present work at SDS/CNTPE

**Working electrodes**	**Linear range (M)**	**Detection limit (M)**	**Reference**
Electrospun carbon nanofibers modified electrode	0.2×10^-6^ -107.0×10^-6^	0.1×10^-6^	5
Carbon paste electrode modified with multi-walled carbon nanotubes enhanced by Sodium dodecyl sulfate	4×10^-7^ -1×10^-4^	5.5×10^-8^	8
Carbon past electrode nanostructures modified gold electrode	3.6×10^-6^ -240.0×10^-6^	1.2×10-5	29
Poly dicyclomine hydrochloride modified carbon paste electrode	2.0×10^-5^- 1×10^-3^	0.638×10^-6^	30
Exfoliated graphene oxide modified glassy carbon electrode	0.5×10^-6^-80.0×10^-6^	0.2×10^-6^	31
Iron(III) Doped Zeolite Modified Carbon Paste Electrode	0.5×10^-6^ -200×10^-6^	80×10^-9^	32
Sodium dodecyl sulfate modified carbon nanotube paste electrode	2.0×10^-6^-5 ×10^-5^	729×10^-9^	This work

### Electrochemical determination of TY at SDS/CNTPE using DPV

**Figure 8:**
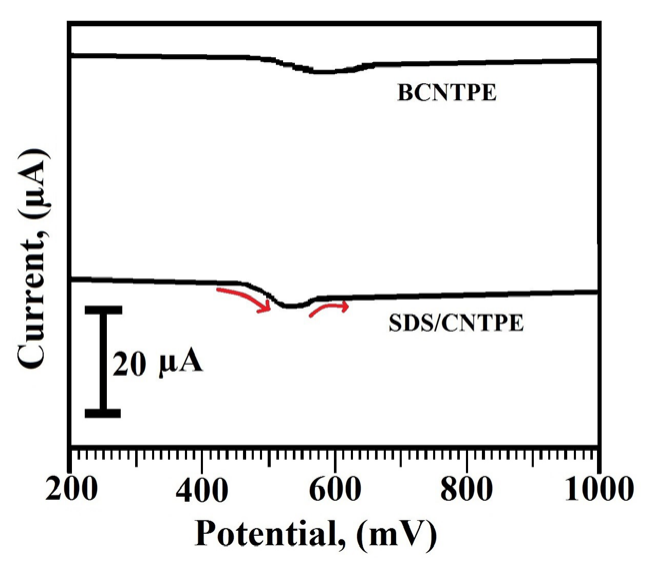
DPV of a solution containing 2×10^-4^ M TY in 0.2 M PBS having pH 7.0 with the scan rate of 100 mV/s at the BCNTPE and SDS/CNTPE

The resolution of TY at SDS/CNTPE was performed using DPV method and it shows superior current sensitivity and improved quality than CV method. Figure 8 demonstrates the voltammogram of DPV for the electrochemical oxidation of 2×10^-4^M TY in 0.2 M PBS having neutral pH with the scan rate 100 mV/s at SDS/CNTPE and BCNTPE. The anodic peak current for the oxidation of TY at SDS/CNTPE was about -39.7 μA, but for BCNTPE the anodic peak current for the oxidation of TY was feeble with the small variation in the peak potential, hence SDS/CNTPE was used as a responsive electrochemical sensor for the sensitive electrochemical investigation of TY.

### Real sample analysis

Thyrobless pharmaceutical drug was used as a real sample for the examination of TY at SDS/CNTPE through CV technique. The detection of concentration and amount of TY was done using standard addition method. The results were tabulated in Table 2, confirming that the SDS/CNTPE shows more consistency, sensitivity, and selectivity towards TY in a real sample as a pharmaceutical drug with the recovery 97% to 98%.

**Table 2:** Determination of recovery of TY in a pharmaceutical drug

**Added (µM)**	**Found (µM)**	** SD**	**RSD (N=3)**	**Recovery (%)**
9.90	9.66	0.1767	0.87	98
13.20	12.90	0.1767	0.87	98
16.50	16.08	0.2121	0.89	97

SD, Standard deviation; RSD, Relative standard deviation; N, number of trials.

## Conclusion

The novel SDS/CNTPE was first used for the responsive determination of TY. The electrode was steady, easy to prepare, easily renewed and low cost, and show high electrocatalytic activity for the oxidation reaction of TY. The Oxidation peak potential was observed at 569 mV with an oxidation peak current of -34.7 μA. This method was irreversible, diffusion controlled with one electron transfer process. The CV and DPV methods showed remarkable sensitivity, selectivity, supplementary response with excellent stability, repeatability, reproducibility towards TY at SDS/CNTPE. The CV method reveals a low LOD of 729 nM and low LOQ of 2.43 µM respectively. The above data concludes that the growth of neurotransmitters, hormones, mental performances, alertness or memory, depression, and high blood pressure in living organisms nearly depends on the catalytic activity of TY. Hence in the case of abnormality of TY, we use TY containing pharmaceutical drugs and foodstuffs for recovery. All this work shows that the electro-analytical scheme was admirable and healthy for the analysis of microscopic electro-active scaffolds.

## Ethical Issues

No ethical issues for this work.

## Conflict of Interest

No conflict of interest with any organization, reviewers and authors for this work.

## Acknowledgments

We gratefully acknowledge the financial support (KFIST) from the VGST, Bangalore under Research Project No: KSTePS/VGST-KFIST (L1)2016-2017/GRD-559/2017-18/126/333, 21/11/2017.
